# Navigating fear, shyness, and discomfort during menstruation in Cambodia

**DOI:** 10.1371/journal.pgph.0000405

**Published:** 2022-06-09

**Authors:** Gabrielle Daniels, Marin MacLeod, Raymond E. Cantwell, Danya Keene, Debbie Humprhies

**Affiliations:** 1 Epidemiology of Microbial Diseases, Yale School of Public Health, New Haven, Connecticut, United States of America; 2 Dalla Lana School of Public Health, University of Toronto, Toronto, Canada; 3 Water, Sanitation, and Hygiene Program, Samaritan’s Purse, Phnom Penh, Cambodia; 4 Social and Behavioral Sciences, Yale School of Public Health, New Haven, Connecticut, United States of America; University of Bremen: Universitat Bremen, GERMANY

## Abstract

While increased attention has been given to girls’ menstrual hygiene management (MHM) experiences in schools as they relate to managerial challenges, research exploring girls’ psychosocial experiences during menstruation and their needs in non-school environments remains limited. This study investigates the knowledge, attitudes, and practices regarding menstruation and MHM (M&MHM) among rural Cambodian girls (at least 14 years old, post-menarche; n = 130), mothers (n = 93), fathers (n = 15), teachers (n = 37; 54.1% female), and boys (at least 14 years old; n = 59) in both the home and school environments. Qualitative and quantitative data were collected through structured interviews, focus groups, and latrine surveys in eight secondary schools and villages from two rural provinces, Banteay Meanchey and Kratie. Findings indicated that although 95% of girls felt capable of managing their menses each month, many girls experienced fear, shyness, and discomfort (FSD) during menstruation. Identified M&MHM challenges and FSD in both the home and school environments influenced all participant groups’ decision-making, social interactions, and varied based on their knowledge of M&MHM and emphasized the need for comprehensive interventions that reduce the impact of MHM challenges on psychosocial experiences and FSD to promote girls’ well-being during menstruation, particularly in income limited settings.

## Introduction

Over the last decade, increasing attention has been given to the disparate impact of inadequate water, sanitation, and hygiene (WaSH) development on women and girls in low- and middle-income countries, whose WaSH needs over their life-course include attending to their menstrual needs and care [1, 2]. Menstrual hygiene management (MHM) has been broadly defined as self-efficacy regarding menstrual care, including access to reliable absorbent materials, appropriate WaSH facilities, hygienic disposal methods, and accurate knowledge of MHM health behaviors [3–5]. Particular focus has been given to adolescent girls’ MHM experiences in relation to their health and educational outcomes [1, 4, 5, 6], and studies consistently support addressing girls’ MHM challenges through improving school WaSH facilities and introducing MHM curriculum [3, 7, 8]. However, less emphasis has been placed on addressing the psychosocial dimensions of MHM and on understanding MHM-related challenges outside the school environment.

Crichton et al. identified psychosocial challenges, such as knowledge deficits, limited social support, and “emotional distress” as underlying contributors to “menstrual poverty” among girls [9]. Crichton et al. concluded that the unnecessary “psychological burden” created by these psychosocial challenges, coupled with managerial WaSH-related concerns, impacted girls’ social and physical well-being, and deserved greater attention in MHM-related research and interventions [9]. Similarly, Rheinlӓnder et al. reported senior schoolgirls from a hygiene and sanitation deprived peri-urban community in Ghana experienced severe hygiene and menstrual poverty on infrastructural, social, and emotional levels [8].

Beyond school settings, research examining MHM challenges in other environments, such as the home, has been limited. Lack of MHM knowledge is cited widely in the literature as a problem for schoolgirls seeking to manage their menses, and access to information in the home setting has been studied in at least two cases. In Mansoura, Egypt, a study of 664 schoolgirls aged 14–18 years found that 24.6% of girls lacked privacy to practice MHM in their homes [10]. Among this group, logistical regression analysis also highlighted that access to mass media in the home, such as radio, was the greatest predictor of sanitary pad use [10]. Consistent with this finding, a study of 250 adolescent schoolgirls in northern Ghana found that girls were more likely to have poor menstrual knowledge if they were from homes without television or radio sets, compared to girls in homes with television or radio sets [11]. In exploring women’s risk for urinary tract infections (UTI) in relation to their ability to practice MHM at home, an investigation in Odisha, India found that home environments with an indoor water supply and convenient places to change enabled women to better practice MHM and reduced their risk for UTIs compared to women who had to change or clean themselves outdoors [12]. Considering that women’s and girls’ ability to navigate their MHM at home may impact their ability to navigate MHM in other settings (e.g., work or school), understanding the factors that impede or enhance MHM in home environments may provide insights into addressing MHM challenges across settings [13].

A systematic review of 76 studies of women’s and girls’ experiences of menstruation in low- and middle-income countries found the majority of MHM studies have been conducted in Africa (n = 45/76) [2]. Yet, interest in investigating menstrual and MHM (M&MHM) experiences across Southeast Asia is growing. Research in this region has included investigating Cambodian schoolgirls’ experiences of MHM in urban and rural school environments. Key findings included an expressed need for more privacy in and around latrines, and provision of sanitary materials and water in latrines; these studies have also noted the need for more MHM and health education, especially prior to onset of menarche [14–16]. Research on MHM in other areas of Southeast Asia have included investigating menstruation practices among school and out-of-school adolescent girls in Lao PDR [17] and a larger survey of MHM-related needs in the East Asia and Pacific regions [18]. While giving particular attention to WaSH-related needs, these studies also confirmed the need to further investigate psychosocial factors impacting women and girls during menstruation.

The present study explored the M&MHM experiences of rural Cambodian girls, with an expanded scope to include the investigation of psychosocial dynamics and managerial challenges in the home and school environments. In recognizing the influence of authority figures and male peers on girls’ M&MHM experiences, parents, teachers, and male students were included in the study to learn more about their roles, responsibilities, and perceptions regarding M&MHM. Central aims of the study included exploring how girls experienced their M&MHM and identifying factors that influenced their M&MHM experiences across environments. Evidence from this study further contextualizes girls’ MHM needs and preferences that can be addressed through governmental or NGO-based interventions to support girls’ ability to manage and care for their MHM as they continue their education and navigate their social environments.

## Methods

**Fig 1:**
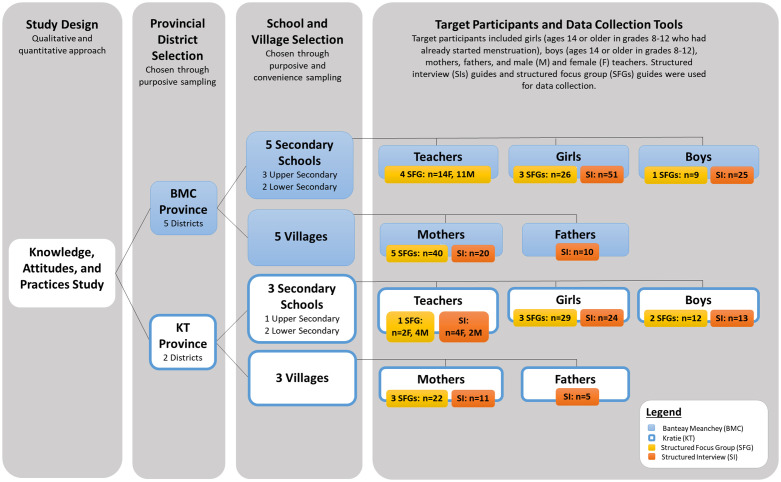
Study design overview. Diagram describing the study design process and final selection of study locations, participants, and participant numbers. Total number of participants: n = 334.

To explore M&MHM among rural Cambodians, a knowledge, attitudes, and practices (KAP), mixed-methods study design was chosen (Fig 1). The primary partner for this study was Samaritan’s Purse (SP) Cambodia, whose interest in MHM and experience with WaSH and Health programming was instrumental to the study design and implementation.

### Ethics statement

Ethical approval for this study was granted from the institutional review board of Yale University’s Human Subjects Committee (HSC Protocol 1506016029; Approval June 12, 2015) and from the Cambodian National Institute of Public Health’s National Ethics Committee for Health Research (236 NECHR, Approval July 3, 2015). All participants provided informed consent prior to enrollment, and all interactions between team leaders and potential participants were conducted in Khmer.

Team leaders reviewed the consent forms verbally in Khmer that explained the study’s topic of MHM with all potential participants prior to receiving their consent. Adults gave verbal consent prior to being interviewed or participating in a focus group. For minors under 18 years of age, they were also provided with a parental opt-out form in Khmer, which was either given directly to the students by the research team or distributed to the students by the school director. Students were asked to discuss the parental opt-out form with their parents. The parental opt-out forms were to either be signed and returned, signifying that the student could not participate, or kept at home as indication of consent.

Team leaders verbally reviewed the study eligibility and purpose with girls and boys who had permission to participate, or who were old enough (18 years old or older) to consent themselves, before beginning the interviews or focus groups. The girls and boys had the opportunity to decline participation at that time or at any point during the conversation due to the sensitive nature of the topic. Only six students returned their parental-opt out forms, indicating a lack of parental consent to have their child participate, and those students were not included in the study.

### Study setting

This study was based in rural Cambodia, specifically in Banteay Meanchey (BMC) province in the northwest and Kratie (KT) province in the northeast. The rationale for working in rural Cambodia was that rural schools and villages may have fewer amenities, in terms of WASH and access to supplies, compared to urban areas, thus presenting unique challenges to rural adolescent girls and women during menstruation. Nationally, at the school level, 54.1% of urban schools (pre-school through college; n = 1,382) lacked a reliable water supply, and 34.7% of urban schools lacked a latrine according to the 2014–2015 Education Statistics and Indicators of Cambodia [19]. Comparatively, of the 745 schools in BMC, 63.5% lacked a water supply, and 38.5% were without a latrine [19]. Likewise, of the 373 schools in KT, 51.7% lacked a reliable source of water and 35.5% lacked a latrine [19]. BMC and KT were suitable because both provinces are within SP’s target programming areas, and SP’s ongoing collaborations with the provincial Ministry of Education, Youth and Sport in BMC and KT were useful in facilitating partnerships with local schools and villages.

Additional details from Cambodia’s 2014 DHS relating to the education and employment of the 15–49-year-old population in the two study populations and the rural population, nationally, are presented in Table 1.

**Table 1:** Overview of education and employment information for the study provinces and rural average.

	By Gender	BMC	KT	Rural (Nationally)	Table number from 2014 DHS [16]
Mean years of education completed	Male	3.9	3	3.9	Table 2.4.2
	Female	2.6	3.4	2.7	Table 2.4.1
Gross secondary school enrollment ratio	Male	53.7	38	48.6	Table 2.5
	Female	52.4	46.7	51.5	Table 2.5
Percent of population that completed primary school (or more)	Male	26.6	64.5	28.4	Table 5.2.2
	Female	44.6	34.3	42.9	Table 5.2.1
Percent of adults working in agriculture	Male	56	69	5	Table 5.6.2
	Female	41	73	52	Table 5.6.1

At the household level, the 2014 Cambodia Demographic Health Survey (DHS) indicated that urban households more often used an improved source of drinking water during the dry season (Urban: 95.0%; Rural: 60.1%) and during the rainy season (Urban: 97.6%; Rural: 81.4%) than rural households [20]. The increase in use of improved water sources during the rainy season among rural households was attributable to an increase in the use of rainwater as a reliable source of drinking water (Dry season: 10.1%; Rainy season: 40.8%) [20]. In terms of latrine coverage, the 2014 Cambodian DHS also indicated that 50.4% of rural households across Cambodia had no latrine or WASH facility coverage, compared to only 6.9% lack of facilities among urban households [20]. Lack of access to a latrine and/or reliable source of clean water would present barriers to appropriate MHM.

Particular to our study’s timeframe is that students were preparing for their final exams. Additionally, previous efforts had been made by the Ministry of Education, Youth, and Sport to distribute “Growth and Changes” booklets describing M&MHM in the local language of Khmer to select schools, some of which participated in our study. At the time, Cambodia’s National Ministry of Education, Youth, and Sport and the National Ministry of Rural Development currently did not have action plans to address MHM at the school or household level, respectively [21].

### Sample and participant selection

Locations for recruitment and data collection were chosen in three stages. Purposive sampling was used to choose five districts in BMC and one district in KT in stage one and to select secondary schools from each of the chosen districts in stage two. Variation in school selection was introduced in the size of schools chosen (i.e., between 170 and 2,217 students) and whether they were lower (usually grades 7–9) or upper (usually grades 10–12) secondary schools. Sampling from schools with large (e.g., 2,217) and small (e.g., 170) student population was intentional to avoid potential bias associated with schools’ size or the likely proximity to large communities. In stage three, a convenience sample of villages were selected from communes (referring to collections of villages) where the students from selected schools lived. Our final study design included five secondary schools and five villages in BMC, and three secondary schools and three villages in KT. A greater number of schools in BMC were included as compared with KT owing to the larger population in BMC, which is 2.3 times that of KT [22].

Our target participants included adolescent girls, mothers, fathers, teachers, and male peers (also referred to as ‘boys’). The eligibility guidelines for girls and boys included being at least 14 years old and attending secondary school. Girls were eligible only if they had already begun experiencing menstruation. Parents were eligible if they were 40 years or older and had at least one daughter who was 14 years old, although the protocol was amended to include younger parents aged 30 years or older so that younger mothers who had traveled long distances, or who were particularly interested in learning from the focus group discussions, could also participate. Secondary school teachers were eligible to participate if they taught in grades 7–12, which overlapped with the grades of participating students.

The consent and recruitment process involved meeting with school directors, commune chiefs, and village chiefs to introduce the study and invite collaboration. After receiving permission to conduct the study, village chiefs were asked to transect their communities and select at least thirteen mothers [nine for structured focus groups (SFGs), four for structured interviews (SIs)] and two fathers for structured interview in their villages per study eligibility criteria. Separately, school directors were asked to recruit teachers and assist with inviting students to participate. At each school, at least fourteen girls and eight boys for SIs and another ten girls or boys for gender-separated SFGs were randomly selected from class lists. All students received consent forms to give to their parents and time to receive parental permission. Only six students opted out of participation by returning the forms. The overall study sample reflected a variety of socioeconomic backgrounds and educational experiences.

We enrolled 334 participants and conducted 165 individual SIs and 22 gender-segregated SFGs (n = 169 participants in SFGs) as detailed in Table 2.

**Table 2:** Participant enrollment and participation details.

Participant group	Number of participants in			Percent breakdown
	Structured interviews	Structured focus group discussions	Total	
Girls	75	55 (in 6 SFGs)	130	39%
**Mothers**	31	62 (in 8 SFGs)	93	28%
Fathers	15	0	15	4%
Female teachers	4	16 (in 5 SFGs)	20	6%
Male teachers	2	15 (in 5 SFGs)	17	5%
Boys	38	21 (in 3 SFGs)	59	18%
Total	165	169 (in 22 SFGs)	334	

### Data collection

Interview guides for SIs and SFGs were developed based on existing MHM literature, input from WASH and Health practitioners, and the theoretical framing of the Social Ecological Model (SEM) [23]. The SEM recognizes that health outcomes have individual, interpersonal, communal, and societal-environmental influences [23], and our interest was in learning how factors at these various levels influenced girls’ experiences of M&MHM. At the individual level, participants were asked about their personal experiences concerning M&MHM, how they navigated those experiences through personal decision-making, and what information and resources they relied on as individuals when navigating issues concerning M&MHM. At the interpersonal level, we examined how various relationships–parent to daughter, teacher to student, peer to peer–impacted the experience and navigation of M&MHM, both in terms of support and in terms of challenges identified. At the communal level, particularly within the context of village communities and school settings, we examined the impact of communal resources and infrastructure on girls’ ability to navigate M&MHM and how each setting influenced girls’ sense of self-efficacy or limitation. At the level of societal-environmental influences, we explored the social, economic, and educational factors that influenced communal experiences, such as access to WaSH resources, economic considerations impacting access to menstrual supplies, and availability of MHM related curricula, to better understand the barriers and supports that these factors introduced for M&MHM. The SI and SFG guides were tailored to each participant group. All questions were finalized after internal review, quality checks, and field testing and were translated into Khmer.

Approval to engage with the communities was sough in the advance from the Provincial Department of Rural Development for both BMC and KT. In addition, approval for activities at the schools was sought from the Provincial Department of Education, Youth and Sport for both BMC and KT. The research team consisted of the primary investigator, two Cambodian women as team leaders, and seven young Cambodian women serving as enumerators, all of whom were trained in study methods to understand the questions and engage participants. No men or young men were included as data collectors and enumerators, as it was decided that it would be beneficial to have Cambodian women who could personally relate to the experiences of having menstruation to discuss the topic with study participants. All SFGs and SIs were conducted in Khmer and involved careful note taking by enumerators. All SFGs, except with teachers, were homogenous based on gender. SIs and SFGs, whether in the villages or schools, were held in private locations chosen by the village chief or school director. For girls and boys, team leaders would privately ask about eligibility and parental consent prior to inviting them to participate. Girls were discretely asked whether they had begun their menstruation to participate, and girls were invited to participate if they verbally confirmed that they had experienced menarche. Demographic information was collected, and SFGs generally lasted one-and-a-half to two hours, while SIs were generally one hour for mothers and girls and 30–45 minutes for fathers and boys. In appreciation of their participation, all participants were offered snacks and/or soap, and a “Growth and Changes” booklet that explains menstruation in Khmer. Some schools participating in our study had already received “Growth and Changes” booklets through prior distribution efforts made by the national Ministry of Education, Youth, and Sport.

During school visits, school directors also assisted the team in completing latrine observational surveys by answering any necessary questions. Additional contextual data was collected in the form of field notes and a brief supplemental pad survey (PS).

### Data analysis

All Khmer transcripts were translated into English prior to analysis. Qualitative data regarding girls’ M&MHM experiences and other participant (parents, teachers, boys) experiences related to MHM was derived from analyzing the SIs and SFGs responses using Dedoose qualitative software. The SIs and/or SFGs with parents, teachers, and boys were analyzed for triangulation purposes, as well. For qualitative analysis, two authors collaboratively developed a code structure using inductive techniques from Grounded Theory [24]. Code structure was developed iteratively following two individual pilot coding exercises, and the final codebook represented themes ascertained both deductively from the literature and inductively based on emerging themes in the transcripts.

Quantitative data regarding girls’ M&MHM experiences and other participant experiences related to MHM was derived from SI responses to close-ended questions that were compiled in Dedoose and exported as tabulated data. Quantitative data concerning latrine conditions in schools was derived from latrine surveys. Quantitative data was analyzed using SAS software, Version 7, to determine frequency measures of medians, averages, and standard deviation for demographic data and response percentages from SI close-ended questions and the latrine data.

### Inclusivity in global research

Additional information regarding the ethical, cultural, and scientific considerations specific to inclusivity in global research is included in the S1 File.

## Results

### Participant characteristics

Each participant group’s demographic data is summarized in Table 3. Notably, among parents with daughters ages six to eighteen years old, nearly 50% reported that they did not have a daughter ages six to eighteen years old still enrolled in school because their daughters had withdrawn early or married at a young age. This is consistent with Cambodia’s 2014 DHS data [20] which reports 51% gross secondary school attendance for the rural female population (52% for BMC and 47% for KT), which suggests that the respondence who are parents were representative of the sample population. It also indicates that the parental responses provide information regarding the adolescent girls and household that no longer attend school.

**Table 3:** Participant characteristics.

Participant Group and Characteristics	
Adolescent Girls (Grades 8–12)	***N = 130*** (% Responded)^1^
Median age (years)	**16** (100.0)
Range (years)	**14–21** (100.0)
No. girls ages 14–17	**101**
No. girls ages 18–21	**29**
*SI responses only*	*n = 75*
Average household size^2^	**5.1 ± 1.6** (100.0)
Average age of menarche (years)	**14.2 ± 1.0** (100.0)
Avg. length of menstruation (days)	**4.4 ± 1.4** (98.7)
Adolescent Boys (Grades 8–12)	***n = 59***
Median age (years)	**17** (100.0)
Range (years)	**14–20** (100.0)
No. boys ages 14–17	**44**
No. boys ages 18–20	**15**
*SI responses only*	*n = 38*
Average household size^2^	**5.2 ± 1.5** (97.4)
Parents (Fathers and Mothers)	***n = 108***
All Parents, % Fathers	**13.9** *(n = 15/108)*
Median number of daughters	**2** (100.0)
Range	**1–6**
Median age (years)	**21** (89.8)
Range (years)	**3–48**
Median number of daughters who have begun menstruating	**2** (100.0)
Range	**1–5**
*Parents with daughters ages 6 to 18 years old only*^3^	*n = 55*
At least one daughter ages 6 to 18 years old still in school (%)	**50.9** (*n = 28/55)*
*Mother responses only*	*n = 93*
Still personally experiencing menstruation (%)	**42.9** (97.8)
Teachers (Men and Women)	***n = 37***
All teachers, % Women	**54.1** *(n = 20/37)*
*SFG responses only*^4^	*n = 31*
No. of teachers with a daughter who has begun menstruation	**1** (100.0)
Average number of years working as a teacher	**6.3 ± 4.2** (100.0)

^1^ Data includes SI and SFG participant responses, unless otherwise indicated. Averages are presented with **±** standard deviation.

^2^ Household size defined as number of members in household.

^3^ Fifty-five parents of all mothers and fathers reported their daughter’s age and/or school status being between the ages of 6 to 18 years old. Six other BMC parents were missing their daughter’s age data, but reported their daughter was not in school.

^4^ Full demographic data was collected for teachers in SFGs; however, there was incomplete demographic information collection for teachers who participated in SIs (n = 6).

The average household size of 5.1 for adolescent girl participants is slightly higher (but not significantly different) than the average of 4.6 household size of 4.6 according to Cambodia’s 2014 DHS data [20], likely owing to the study focus of including households with children.

### Perceived self-efficacy for MHM

Similar to prior studies, girls in this study identified various resources and challenges that impacted their M&MHM experiences. When girls were asked if they felt capable of successfully managing their M&MHM each month, 94.7% (n = 71/75) of girls reported feeling capable despite the managerial challenges they described. Common reasons for self-efficacy included having access to supplies and facilities, practical MHM knowledge, social support, and repeated experience over time:

*[I can manage] because [my] parents often tell* [teach] *me*, *and I clean [my] vagina well*. *I am able to know about menstruation and what to do for well hygiene*, *and [I] have enough sanitary pad[s]*.

(Girl SI, KT, Age 15, Grade 8)

*[I can manage] because [I am] used to experience before and no longer feel shy*. *[I] have sanitary pad ready*, *have guidance from friends and teachers about menstrual management related to food*…*etc*.

(Girl SI, BMC, Age 16, Grade 9)

*[I can manage] because I learned about menstruation many times and [have] experience[d] [it] frequently*. *[I] can prepare myself for supply all the time*.

(Girl SI, BMC, Age 18, Grade 12)

Even with their confidence, girls also described feelings of fear, shyness, and discomfort (FSD) in multiple aspects of their M&MHM experiences (Table 4). When asked how girls generally felt during menstruation, 65.3% (n = 49/75) of girls indicated feeling scared and/or shy. Another 17.3% (n = 13/75) described feeling burdened, uncomfortable, or generally unhappy during menstruation.

**Table 4:** Girls’ reports of self-efficacy and feelings during menstruation.

**Self-Efficacy**	***N = 75 (% Responded)*** ^1^
Feels capable of managing menses each month	**94.7%** (100.0)
**Feelings about Menstruation**	*N = 75* (% Responded)^2^
Normal	**12.0%** (100.0)
Grown Up	**8.0%** (100.0)
Scared and/or Shy	**65.3%** (100.0)
Burdened, Uncomfortable, Bored	**17.3%** (100.0)
Happy and/or Excited	**4.0%** (100.0)

^1^ Specifically asked: “How do you feel about experiencing menstruation and why?” The question was purposefully open to capture general feelings about experiencing menstruation, regardless of the timing or environment. Girls could give multiple responses

^2^ Analyzed responses are from girls’ structured interviews only.

As girls described their experiences during M&MHM, feelings of FSD were often named together. For example, when a group of girls from KT were asked during a SFG, “Can you explain how girls feel about having their menstrual cycle occur at school?”, the girls participating (GP) in the SFG gave the following responses:

*Feel shy*.

(GP1, Age 18, Grade 11)

*Scared*, *shy*, *and afraid of being known*.

(GP2, Age 17, Grade 11; GP3, Age 15, Grade 8; GP4, Age 15, Grade 9; and GP5, Age 16, Grade 8)

*Bored because afraid of being known*.

(GP6, Age 17, Grade 10)

*Feel uncomfortable*, *shy*, *and afraid of being stain[ed]*.

(GP7, Age 16, Grade 10; and GP8, Age 17, Grade 10)

*Scared*, *feel uncomfortable*, *afraid of being stain[ed] and known*.

(GP 9, Age 15, Grade 8)

Many girls described experiencing FSD at the onset of menarche, noting that they were unprepared and felt scared and/or shy because they had “never known” or understood menstruation beforehand. As one girl described:

*At school*, *I did not realize [it was menarche]*, *but my friend told me*. *And I was scared and ask permission from teacher going home*. *When I arrive home*, *I dared not to tell my mother*. *My mother wondered and kept asking me*. *I told her that I bled*. *My mother bought me disposal pad*, *and she told me that this is menstruation*.

(Girl SI, BMC, Age 16, Grade 8)

Some girls described experiencing less FSD over time after menarche. However, many girls reported experiencing some degree of FSD as an ongoing part of their menstrual experiences, particularly while at school. FSD was often related to MHM challenges that increased the likelihood of menstrual accidents (e.g., leakage, staining) or being viewed as unhygienic, as three girls described when asked how they felt about experiencing menstruation at school:

*[I feel] shy sometimes [when] I don’t wear sanitary pad properly and afraid of being stain the skirt*. *And sometimes I dare not to go out the class*. *If I want to eat*, *I ask my friends to buy*. *I sit always and dare not to play; [I] always feel bored*.

(Girl SI, BMC, Age 14, Grade 8)

*[I] feel shy and afraid of bleeding out on chair*. *I don’t like to talk to someone*, *and I like to be alone*. *If I got blood stain*, *I will be embarrassed and do not know how to do*. *I do not [want] to talk to someone if they question me*, *and I do not know how to answer*.

(Girl SI, BMC, Age 15, Grade 8)

*[I] feel difficult and shy because sometimes menstruation comes without preparing sanitary pad*, *[and] it can be stained out*. *Sometimes menstruation comes a lot until staining out the skirt*.

(Girl SI, BMC, Age 18, Grade 12)

### Managerial experiences and implications for MHM

Girls emphasized the importance of being able to comfortably practice MHM at home and at school to avoid FSD related to menstrual accidents, hygiene concerns, and pain and discomfort management during menstruation. All girls reported using disposable sanitary pads for their MHM but would also use non-reusable handkerchiefs or napkins if they did not have a sanitary pad. During menstruation, 66.7% (n = 72) of girls preferred to change their sanitary pads three or more times a day, and girls reported preferences of wanting to clean themselves, including showering, at least two times per day. Girls also described the value of having pain relief medicine during their menstruation, as at least 43.7% (n = 71) of girls reported experiencing pain or discomfort (PD) “sometimes” during menstruation and 14.1% (n = 71) reported “always” experiencing PD during their menstruation. To comfortably manage their MHM, girls placed value on having privacy, reliable menstrual products, and described wanting at least four key WaSH resources for their M&MHM practices, including (1) an acceptable latrine, (2) clean water, (3) cleaning supplies, including soap, tissue, or towels, and (4) non-embarrassing disposal options. However, girls reported that barriers existed in accessing these needs and resources, particularly the four key WaSH resources, in both the home and school settings (Table 5).

**Table 5:** Managerial practices and experiences during menstruation.

Practices and Experiences	
Type of absorbent material used^1^	N = 75 (% Responded)^2^
Disposable pads	**100.0% (100.0)**
How many times girls changed sanitary pad or cloth per day^3^	N = 75 (% Responded)^2^
Two to three times	**33.3% (96.0)**
Three or more times	**66.7% (96.0)**
How often experiencing pain or discomfort during menstruation	N = 75 (% Responded)^2^
Always	**14.1% (94.7)**
Often	**25.4% (94.7)**
Sometimes	**43.7% (94.7)**
Rarely	**8.5% (94.7)**
Never	**8.5% (94.7)**
How girls accessed supplies	N = 75 (% Responded)^2^
Receive from family member	**33.3% (96.0)**
Receive from friend	**1.4% (96.0)**
Receive money from home to purchase	**70.8% (96.0)**
Supplies provided at school	**2.8% (96.0)**
Make them at home	**5.56% (96.0)**
Household latrine conditions, girls report	N = 75 (% Responded)^2^
Has access to a comfortable toilet/latrine to use during menstruation	**81.1% (98.7)**
Has access to an area for washing stains or bathing	**88.0% (100.0)**
Has access to clean water for washing hands	**66.7% (100.0)**
Has privacy when using the household latrine	**66.7% (100.0)**
Has access to a private place for disposing sanitary pads or supplies	**72.0% (100.0)**
Has access to soap	**92.0% (100.0)**
School latrine conditions, girls report	N = 75 (% Responded)^2^
Has access to a place to dispose of used sanitary materials	**18.7% (100.0)**
School latrine survey conditions	N = 28 latrines (no./N)^4^
No. of clean latrines	**35.7% (n = 10/28)**
No. of latrines with soap inside or nearby	**25.0% (7/28)**
No. of latrines with private trash bin or nearby private disposal option	**0.0% (0/28)**
No. of latrines with door that locks from the inside	**89.3% (25/28)**
No. of latrines with door that allowed “peaking” (e.g., holes in door)	**53.6% (15/28)**

Girls reported on various managerial practices and experiences during their menstruation, including access to supplies, WaSH facilities and resources, and pain and discomfort management.

^1^ Choices included disposable pads, reusable pads/cloth material, tissue, and other. Girls could choose more than one option.

^2^ Analyzed responses are from girls’ structured interviews only. N = 75 girl SIs.

^3^ No girl reported changing pads or sanitary cloths only one time per day.

^4^ At all eight participating schools, the latrines on the campus grounds were surveyed. Although some latrines were locked and not opened during school visits, twenty-eight latrines were able to be observed that were also available for use by students. Notes were taken qualitatively to document the condition of the latrine and converted into count data.

Lack of privacy, MHM supplies, disposal options, clean water, and acceptable latrines created MHM challenges for the girls. These challenges resulted in girls having to make difficult decisions related to M&MHM practices and their available resources:

*[I] bring sanitary pad from home; [I do] not change at school because toilet is not clean and dare not to throw*. *When I need to change*, *I go home*.

(Girl SI, BMC, Age 15, Grade 10)

*My friend does not have enough water*. *I saw her pants was blood stain[ed] because she did not put pad*. *She did not have money to buy [a] pad*. *[She has] no toilet at her house*, *and she used [the] toilet of her neighbor*.

(Girl SI, BMC, Age 18, Grade 8)

#### MHM in the home environment

In general, girls reported having better access to MHM-related resources at home compared to their access to MHM-related resources at school. At least 81.1% (n = 74) of girls reported that they had access to a comfortable toilet/latrine to use during menstruation at home, and 88.0% (n = 75) of girls said they had access to an area for washing stains or bathing. Over 60% of girls reported having access to clean water for handwashing, privacy when using the household latrine, private disposal options, and nearly all girls (92.0%, n = 75) reported having access to soap for cleaning. To access MHM supplies, such as sanitary pads, 33.3% (n = 75) of girls reported receiving supplies from family members and 70.8% (n = 75) of girls reported receiving money from home to access supplies compared to only 2.8% (n = 75) of girls who reported that they accessed MHM supplies at school. Girls often expressed preferring, or having fewer barriers, to manage their MHM at home based on the available resources and increased privacy, both of which resulted in less FSD, as two girls described:

*[I] feel scared because [school] it is not home*. *Because at home*, *there are not many people*, *and I have sanitary pad*. *But I am afraid of being seen [with a] blood stain at school*.

(Girl SI, BMC, Age 16, Grade 10)

*[At home*, *I] take a shower in the morning*, *two times per day*, *and change sanitary pad properly*. *At home*, *there is private [privacy]; [I am] not shy and have enough supply*.

(Girl SI, BMC, Age 20, Grade 12)

For some girls, however, household or village WaSH resources and other MHM supplies were sometimes inadequate or unavailable. According to Cambodia’s 2014 DHS data, nearly 50% of rural households lacked latrines [20]. Lack of clean water was a significant concern for girls, who reported being unable to clean themselves properly during menstruation or had concerns about the risk of infection from unclean water. Although most girls (72.0%, n = 75) reported having a private place for disposing sanitary pads or supplies at home, girls described that these disposal options were not always convenient or easily accessible. Girls also reported financial barriers to having MHM-related supplies, such as sanitary pads and medicine for PD relief, while at home. When asked what challenges they or other girls faced when practicing MHM in their homes or villages, several girls described these challenges in the following ways:

*[Girls] lack medicine [and it can be] difficult to clean*. *They don’t know about hygiene*. *[It is] hard [to] find the place for disposal; clean water is not enough*, *and toilet is not clean*.

(Girl SI, BMC, Age 14, Grade 9)

*During raining season*, *[it is] difficult to bury sanitary pad*. *The sanitary shop is far to buy; most people have no toilet*, *lack clean water*, *and [it is] hard to get medicine when [having] stomach pain during menstruation*.

(Girl SI, BMC, Age 16, Grade 9)

*[I] lack financially and time to care my body during menstruation*. *[I] feel stomach pain and lack medicine to release pain*.

(Girl SI, KT, Age 16, Grade 10)

*[I] do not take [a] shower in the pond because [I am] afraid of being transmitted [a] virus from unclean water*.

(Girl SI, BMC, Age 17, Grade 10)

*Sometimes*, *the girls lack money to buy sanitary pad*, *lack toilet for changing it*, *lack understanding of menstruation*, *and [it is] hard to find place for disposal*.

(Girl SI, BMC, Age 17, Grade 11)

Girls reported the importance of having access to these resources in the home setting to reduce the risk of staining and odors later at school, which increased FSD. As one girl described, “Before coming to school, [I] need to take shower [to] avoid the smell at others” (BMC, Age 16, Grade 10).

#### MHM in the school environment

Once in the school environment, girls reported experiencing FSD and MHM challenges due to limited MHM-supportive resources, including an acceptable latrine and access to clean water. Only 18.7% (n = 75) of girls reported during SIs having access to a private disposal option for their used sanitary materials at school, and all girls who were interviewed reported not being able to access to menstrual supplies, such as sanitary pads, at their school. During the school latrine observation surveys, only 35.7% of latrines were found to be clean, which included not having cobwebs on the walls, no mud tracked over the floor and latrine seat, and lack of water covering the floor. Although 92.9% of latrines had water in the basin for flushing, this was often unclean and not suitable for washing hands, let alone for cleaning the body. However, even if water was available, only 25% of latrines had soap, and no latrines had a trash bin inside for disposing materials. Overall, 53.6% of latrines had doors with broken locks or holes that reduced privacy, and two of the eight schools visited said that the paths to latrines would flood during rainy season, further complicating girls’ experiences of using the latrines during menstruation.

If a girl came to school unprepared for her menstruation or experienced a menstrual accident (i.e., leakage, staining) while at school, the lack of access to a clean place to change, clean water, and emergency menstrual supplies contributed to many girls experiencing FSD, social anxiety, and often choosing to leave school to return home. Lack of private disposal options contributed to some girls resorting to practices such as wearing pads throughout the school day without changing or keeping their used pads with them at school rather than be “seen” trying to throw them away. The practice of prolonged sanitary pad use increased discomfort and FSD due to the possibility for odors and vaginal irritation, as girls sometimes described feeling “itchy” until they were able to clean themselves and change. When girls were asked how they managed their MHM at school, several girls described these challenges and the FSD they experienced, saying:

*[The] school lacks soap*, *clean water*, *sanitary pad*, *room for female students*, *and place for disposal of sanitary pad*.

(Girl SI, KT, Age 15, Grade 9)

*[I] wear sanitary pad from home and wait until the class finishes to change at home*.

(Girl SI, KT, Age 16, Grade 8)

*[At school*, *I] feel scared because I don’t know where I [will] change and how I [will] throw sanitary pad*.

(Girl SI, BMC, Age 18, Grade 11)

*[At school*, *I] lack sanitary pad*, *clean water*, *hygiene*, *[and] room for women to change sanitary pad*. *And [there is] no medicine for women who have menstrual problem*.

(Girl SI, BMC, Age 18, Grade 11)

*[I] must be careful and can’t change*. *I need to use same sanitary pad because there is no toilet to change*.

(Girl SI, BMC, Age 19, Grade 12)

*At school*, *I feel shy*, *lack clean water*, *toilet*, *soap*, *and sanitary pad during menstruation*.

(Girl SI, BMC, Age 20, Grade 12)

Overall, both villages and schools at times lacked important WaSH resources that influenced how girls could manage their MHM in each environment and contributed to FSD, though girls reported having easier access to resources in their home environment. These communal level challenges reflected the societal level challenges that rural households and schools in these districts faced in providing sufficient coverage of clean water and latrines that are key resources for girls during their menstruation.

#### Challenges with sanitary pads and implications for FSD

For comfortable MHM, having reliable menstrual products was reported as a factor that could reduce FSD about leakage and staining. However, girls reported that poor adhesive, inappropriate sizing, limited absorbent capacity, and failure to prevent odors made some pad brands unreliable. When asked specifically about what caused staining-accidents, girls indicated that these unreliable features of pad design, as well as ill-fitting underwear, were reasons for leakage and even impacted how they considered transit between locations:

*[I] should not walk far*, *sit [for a] long [time]*, *[or] ride motor to market because [this] can get us wet or cloth stain that people can see*.

(Girl SI, KT, Age 15, Grade 8)

*The pads turn over for [the] sticker isn’t good*. *The pads are thin and short*, *[and there is] too much blood*. *Accidents happen especially when sleeping and playing*.

(Girl PS, BMC, Age 18, Grade 11)

Although the girls said better brands existed, girls agreed that difficulties accessing better quality sanitary pad were often financial.

All girls reported in SIs that they had trouble accessing menstrual supplies, like pads, at school. When girls were asked what they did if they came to school unprepared and began their menstruation, girls described different approaches to addressing the challenge of not having sanitary pads with them:

*[I] ask friends to buy sanitary pad and ask them to get skirt*. *If [it is an] exam day*, *I will not go home*, *but if not*, *I ask permission to go home*.

(Girl SI, BMC, Age 15, Grade 8)

*[I] ask [my] teacher to go home early or sit still without getting out*. *[I] wait until everyone [is] gone and I leave after*.

(Girl SI, KT, Age 15, Grade 9)

*[I] have friends walk behind [me to] not let people know [there is a stain]*, *and [I] use [a] sweater to tie [it around my] waist*. *[I] ask [my] friend*, *who lives near school*, *for [a] new skirt to wear home*.

(Girl SI, BMC, Age 16, Grade 9)

Even though the girls described being resourceful, the implication was that a lack of emergency supplies available at school limited girls to seeking assistance primarily from other female peers, or, occasionally, female teachers. As girls described, if relational support was available, girls would ask their female peers to help them get sanitary pads. If this was unavailable, girls would occasionally have the resources to purchase their own. However, for many girls, the remaining option was to simply return home.

### Knowledge-related influences on FSD

Having knowledge of M&MHM was influential in girls’ perceived self-efficacy and FSD. Girls generally understood that menstruation involved monthly periods of bleeding and that hygienic MHM was important. Girls also associated menstruation with their process of maturing, and some girls indicated having heard about biological reasons for menstruation.

However, some girls reported having no prior knowledge of menstruation before menarche. Without prior knowledge, several girls reported experiencing fear and confusion before learning about M&MHM at the onset of menarche:

*When I was grade 8*, *at home*, *I got up to the toilet and see the blood from vagina*. *I was scared with thought of disease*. *I asked my mother*, *and she said that this is menstruation*.

(Girl SI, BMC, Age 18, Grade 12)

If girls had knowledge of M&MHM before or immediately at the onset of menarche, girls felt more confident about caring for themselves during menstruation, which alleviated FSD. Girls learned about M&MHM from a variety of sources, but most preferred learning from booklets (45.3%, n = 34/75) or from a female relative (40%, n = 30/75) due to privacy and personal comfort (Table 6).

**Table 6:** Girls’ preferences for learning about M&MHM.

	*N = 75* (% Responded)^1^
Prefers to Learn From ^2^^,^ ^3^	
Booklet (%)	**45.3** (100.0)
Teacher (%)	**30.7** (100.0)
Mother or female relative (%)	**40.0** (100.0)
Nurse or health worker (%)	**8.0** (100.0)

Though learning from books and female relatives was most preferred, some girls were comfortable learning from teachers and a few from a nurse or health worker.

^1^ Analyzed responses are from girls’ structured interviews only. Percentages may sum >100.0% because girls reported multiple options.

^2^ Specifically asked: “How do you prefer to learn about M&MHM, and why?” Response rate: 100.0%.

^3^ An additional option for “drama” was asked, but no girl chosen drama as a preferred learning method.

Most girls had knowledge of MHM practices, such as showering and cleaning with soap; using pads to prevent staining; and changing pads multiple times per day. However, a meaningful gap in knowledge for some girls was the correct way to wear disposable pads, such as knowing how to properly position pads or keep them in place. This lack of knowledge was said to contribute to staining and FSD:

*[I feel] shy sometimes*. *I don’t wear sanitary pad properly and afraid of being stain the skirt*. *[*…*] Sometimes I dare not to go out the class*.

(Girl SI, BMC, Age 14, Grade 8)

*[We] feel scared*. *We wear sanitary pad improperly [and] stain the skirt*.

(Girl SI, BMC, Age 15, Grade 9)

Girls also referenced dietary knowledge as an important area of menstrual knowledge and desired to know more about foods that were beneficial during menstruation. Foods considered to be menstrual-friendly were generally non-sour fruits and green vegetables, whereas sour, spicy, and fermented foods were thought to cause menstrual problems, such as irregular menstruation, odors, and stomach pain.

When girls were asked if they wanted to learn more about M&MHM, 98.7% (n = 74/75) said yes (Table 7). Among those who answered yes, only 24.0% (n = 18/75) of girls were interested in learning more about the biology of menstruation, whereas 44.0% (n = 33/75) were interested in how to improve hygiene. Girls’ responses suggested that pragmatic knowledge of M&MHM was particularly of interest.

**Table 7:** Girls’ interests in learning more about M&MHM.

	*N = 75* (% Responded)^1^
Want to Learn More?^2^	
Yes (%)	**98.7** (100.0)
Would Like to Learn More About^3^	
Basic biology of menstruation (%)	**24.0** (100.0)
How to improve hygiene (%)	**44.0** (100.0)
Proper disposal methods (%)	**29.3** (100.0)
Infections related to poor MHM (%)	**48.0** (100.0)
How to teach others about MHM (%)	**21.3** (100.0)

Almost every girl (98.7%) expressed wanting to learn more about M&MHM. Girls had the most interest in learning how to improve hygiene (44.0%) and avoiding infections due to poor MHM (48.0%).

^1^ Analyzed responses are from girls’ structured interviews only. Percentages may sum >100.0% because girls reported multiple options.

^2^ Specifically asked: “Would you like to learn more information about MHM?” Response % may add to > 100.0% because girls could give multiple responses. Response rate: 100.0%.

^3^ Specifically asked: “If yes, what type of information?” The options were as listed in the table, plus an additional option for other. Response % may add to > 100.0% because girls could give multiple responses. Response rate: 100.0%.

### Social factors influencing FSD

Girls identified several social and relational factors that influenced FSD during menstruation. Altogether, 93.3% (n = 70/75) of girls said they had support from family and/or female peers at menarche, and maternal support was key to navigating FSD and MHM, both at menarche and beyond. Generally, girls reported experiencing less FSD during menstruation around female relatives (i.e., mothers), peers, and teachers compared to male relatives (i.e., fathers), peers, and teachers. Even with these general preferences, relationships that enabled girls to better navigate MHM and helped alleviate FSD were considered supportive and preferable to girls during menstruation. However, girls often reported negative attitudes towards crowding or having to engage with others, particularly male peers, during menstruation, often due to FSD about menstrual accidents or desires for privacy.

#### Girls’ perspectives on maternal support

Girls frequently said their mother was their primary confidant at menarche, and 91.5% (n = 65/71) of girls felt comfortable talking with their mothers about personal health issues. At the onset of menarche, girls said mothers routinely explained that their bleeding was normal, introduced MHM practices, and were the first to provide sanitary pads:

*[I] sat at home*, *[and] immediately I [*…*] [felt] wet and scared when I [*…*] [saw] the blood*. *I ran to tell my mother*, *and she said not to [be] scared because it is normal for mature women*. *Then she told me how to use sanitary pad*.

(Girl SI, BMC, Age 15, Grade 8)

Beyond menarche, even when girls indicated increased self-sufficiency during menstruation, mothers were often still regularly involved in helping their daughters to access supplies and in sharing information regarding MHM:

*[I can manage] because when it is near menstruation*, *I told my mother*, *and she purchases [*…*] sanitary pad[s] [for me]*. *Sometimes*, *my mother has sanitary pad[s]*, *so she gives [to] me*.

(Girl SI, KT, Age 15, Grade 9)

*[I’ve] got to love myself*. *I think that mother[s] can’t help us all the time*. *[I] have enough supply*. *If not*, *[I] ask mother to buy*.

(Girl SI, BMC, Age 18, Grade 8)

Some girls, however, described maternal uncertainty or confusion at the onset of menarche:

*[My] first menstruation [was] at the field*, *and I told my mother*. *She said let’s wait to see next month because she thought that the leech suck during transplantation [a farming practice]*. *A few days later*, *she realized that I experienced menstruation*.

(Girl SI, BMC, Age 18, Grade 12)

When this occurred, maternal uncertainty or confusion contributed to increased FSD for some girls, who thought they were becoming ill rather than experiencing a normal menstruation.

#### Girls’ perspectives on paternal support

Very few girls directly named their fathers as a primary support to them during menstruation. Girls often referred to their “parents” helping to provide money for supplies but rarely noted other paternal involvement during menstruation. Occasionally, girls discussed their father in terms of their interactions with them during menstruation. Some girls noticed little change in their relationship with their father after menarche, as two girls described:

*Before menstruation*, *my father and younger brother always play and go somewhere with me*, *and now it is still the same*.

(Girl SI, BMC, Age 14, Grade 8)

*Before*, *my father brought me outside*, *but now he knows that I am having menstruation*. *He still bring me*.

(Girl SI, BMC, Age 17, Grade 11)

These girls did not indicate experiencing FSD with their fathers during menstruation. Other girls reported changes in their father’s responses to them post-menarche and during menstruation:

*Before*, *[when] my father went somewhere*, *he took me*. *But [when] I experience menstruation*, *he stops taking me because he thinks I grow up*.

(Girl SI, BMC, Age 15, Grade 8)

*[My] father plays with me when I have not experienced menstruation*, *but when I experience*, *he does not allow me to follow him*.

(Girl SI, BMC, Age 18, Grade 11)

The girls did not indicate how these changes in response impacted them during menstruation, however. Likewise, these relational changes may have corresponded with fathers modifying their roles or support to girls during menstruation in other ways, such as helping provide supplies.

#### Girls’ perspectives on teachers’ support

When asked about the school environment, girls expressed mixed attitudes towards teachers during menstruation. Female teachers were often preferred for social support at school due to their personal experience of menstruation, but some girls found male teachers supportive, as well:

*[Female teachers] can help because they have experienced menstruation earlier than us*, *and they have the knowledge earlier than us [and are] able to teach us*.

(Girl SI, KT, Age 16, Grade 9)

*Female teachers are helpful because they let my friends to accompany me home*. *It is hard to ride a bike*. *[They understand] because they have experience before us and [are] same [gender as] us*.

(Girl SI, BMC, Age 16, Grade 9)

*[A] male teacher or female teacher can teach about menstruation and body hygiene during menstruation*.

(Girl SI, KT, Age 16, Grade 8)

*Both male and female teachers [can help]*. *He is able to know the girl[‘s] needs*. *Female teachers experience menstruation*. *She knows about lot menstruation*.

(Girl SI, BMC, Age 19, Grade 12)

Girls said teachers would sometimes help provide pads, teach about M&MHM, and give them permission to rest or care for their MHM needs. Girls particularly appreciated when teachers would allow them to go home early during menstruation if needed, as teachers’ willingness to allow them to leave school reduced FSD. Girls described these supports, stating:

*Teachers can help because they don’t want us to be embarrassed*.

(Girl SI, KT, Age 16, Grade 9)

*They [teachers] can help [to] give the idea [and] provide supply if we tell them*.

(Girl SI, KT, Age 17, Grade 10)

*Teachers are helpful because they let me rest*.

(Girl SI, BMC, Age 18, Grade 12)

*[I] tell teachers and ask them to go home*. *It helps me not to feel scared*.

(Girl SI, BMC, Age 20, Grade 12)

However, some girls did not view teachers as supportive, finding them to be “too busy” or unable to provide supplies. Girls also expressed feeling shy or uncomfortable telling teachers about their menstrual concerns. Girls shared their concerns, stating:

*[Teachers] can’t help because they have nothing to help me*.

(Girl SI, KT, Age 15, Grade 8)

*Teachers are not helpful because they are busy to teach and [have] no spare time*.

(Girl SI, BMC, Age 15, Grade 8)

*[Teachers] can’t help because they don’t bring sanitary pad along with them*.

(Girl SI, BMC, Age 18, Grade 12)

*Teachers are not helpful because students don’t tell teachers*, *and they* [girls] *feel shy*.

(Girl SI, BMC, Age 19, Grade 12)

Taken together, girls’ feedback suggested that their dependence on teacher support was limited and was at times influenced by FSD and lack of managerial supports that the school did not provide, or that individual teachers could not provide.

#### Girls’ perspectives on female peers’ support

Female peers were frequently associated with being supportive and reducing FSD. Girls said their female friends were comforting because they also experienced menstruation, expressed concern for one another, helped each other with supplies, and were considered reliable confidants. Girls assisted one another when accidents or unexpected menses occurred at school, particularly to help hide or prevent stains. When girls wanted to return home, their female friends would also help ask teachers for permission to leave school and at times, accompany each other home. Of these supports, girls stated:

*Friends can help because they don’t want me to [*…*] [feel] difficult during menstruation*.

(Girl SI, KT, Age 15, Grade 8)

*[I feel] shy at people [if] someone talks behind when I [go to] change [my] sanitary pad at home*. *[I] ask friends [to] change [the] table if the stain [is] there and ask them to hide it*.

(Girl SI, BMC, Age 15, Grade 10)

*[If I have an accident*, *I] feel scared and ask friends to purchase sanitary pad*.

(Girl SI, KT, Age 16, Grade 9)

*[I] talk to friends*, *[and] they can help to accompany me home*. *They help to buy medicine*, *find the shirt to hide behind*, *and remind me not to play much because afraid of being seen*.

(Girl SI, KT, Age 17, Grade 10)

However, girls also faced limitations in helping each other, sometimes due to lack of knowledge or supplies, or being too busy with their studies:

*[I] talked to [my] younger god-sister*, *who learns with me*. *She can’t help because she has no sanitary pad with [her]*. *[She] can’t send me home [because she] [*…*] goes [a] different way*.

(Girl SI, BMC, Age 18, Grade 10)

*[I] talked to friends*, *and sometimes they can help; sometimes they can’t help because we have same experience and issue*. *Some friends are busy to study*, *[so] they can’t help*.

(Girl SI, BMC, Age 19, Grade 11)

Given these limitations, it would help girls to have access to other resources at school (e.g., emergency supplies) to help alleviate FSD in times of menstrual emergencies and to better support girls. In the absence of these resources, girls tried to work together to help preserve each other’s dignity and prevent FSD.

#### Girls’ perspectives on male peers’ support

Although some girls expressed indifference towards male peers during menstruation, nearly all girls experienced increased FSD around male peers due to concerns about staining and keeping their menstruation private. Compared to other social contexts, girls emphasized their avoidance of male peers during menstruation often depending on their FSD due to staining:

*[I feel] shy at them* [boys] *because [of] blood stain during menstruation*. *However*, *I am not shy if there is no blood stain*.

(Girl SI, BMC, Age 17, Grade 11)

Coupled with concerns about staining, the girls’ feelings of shyness and embarrassment of experiencing their menstruation around male peers were often associated with and heightened by fears of being discussed negatively or teased. Girls described these concerns, saying:

*[I feel] shy because they are boys [who] never experience menstruation*. *[I am] afraid of being seen [with] the stain*, *and they [*…*] [say] that we don’t know how to clean our body*.

(Girl SI, BMC, Age 14, Grade 8)

*[I] want to hide*, *and [I feel] shy because afraid of being known*. *I am not closed to boys*. *Afraid of being smell at people*, *being stained*, *and [that] people [will] talk from mouth to mouth about this*.

(Girl SI, BMC, Age 15, Grade 10)

*[I] feel shy and [am] afraid that the boys talk to others*.

(Girl SI, KT, Age 16, Grade 8)

For these reasons, girls valued not being “seen” or “known” to be experiencing their menstruation at school. While some girls described that their male peers were not particularly bothersome to them during their menstruation, many girls described experiencing FSD around male peers even if their male peers were unaware of girls’ menstruation. Only one girl said she felt that male peers recognizing stains was a good thing, as they could alert the girl about the issue. However, this was not considered a support to most girls, who described increased FSD under those circumstances.

### Other perspectives on girls’ M&MHM experiences and FSD

The perspectives of mothers and fathers (parents), female and male teachers, and boys (male peers) frequently agreed with girls about the supports they needed during menstruation and discussed varying levels of awareness of some of the M&MHM challenges girls faced. Overall, parents, teachers, and male peers generally expressed concerns about the challenges girls faced during the menstruation and often described wanting to be supportive, though at times, it was unclear to them how they could help girls experience less FSD and enhanced MHM.

#### Perspectives of mothers

Mothers acknowledged that lacking a source of clean water and an available latrine presented several challenges to their daughters. Mothers also said that access to supplies could be a challenge in the home, contributing to FSD. Despite this, some mothers would explain that even if they lacked supplies, their daughter would have enough:

*[There are challenges because] menstruation comes irregularly and lack [of a] toilet makes [it] difficult to change sanitary pad and shower because [there is] no place to hide*.

(Mother, BMC)

*[I] lack supply frequently and find napkin instead*, *[but my] daughter has enough*.

(Mother, BMC)

The majority of mothers generally agreed with the roles and responsibilities girls described during menstruation. Most mothers said they felt confident and comfortable assisting their daughters with menstrual issues, including sharing knowledge, providing supplies, and advising their daughters about rest and diet during menstruation. Several mothers recognized their daughters’ FSD at the onset of menarche or thereafter, and shared their efforts to be supportive to their daughters, as one mother described:

*I advised her not to [be] scared*, *[that] nothing happen[ed] because mature people bleeding is normal*, *and it is menstruation*.

(Mother, BMC)

A few mothers expressed not feeling as needed during their daughter’s menstruation because the daughter had learned how to care for her own MHM. Others found it difficult to support their daughter, particularly at menarche, due to FSD:

*[I] never talk [about menstruation] because my daughter knows herself*.

(Mother, BMC)

*I did not tell my daughter because she hid about menstruation*. *My daughter feels shy to tell about menstruation*.

(Mother, BMC)

Mothers also described having experienced FSD at their menarche with their mothers. This generational pattern of girls hiding menstruation from their mothers thus impacted maternal awareness at the time of menarche.

When asked whose responsibility it should be to teach girls about MHM, mothers generally felt they should be responsible, as well as other female relatives depending on the circumstances. Mothers emphasized wanting their daughters to know more about MHM, diet, and particularly, how to wear sanitary pads properly:

*Mother is responsible to tell because she first experience[ed] [menstruation] and [is] able to tell daughter about menstruation*.

(Mother, BMC)

*Only older sisters can teach [girls about MHM] because no one stays at the village at daytime*. *They go to work and come back at night*.

(Mother, KT)

*[I would like my daughter to have] the information related to food [she] can eat during menstruation and using sanitary pads*.

(Mother, BMC)

*[I] would like my daughters [to] know about hygiene through people coming to teach or from different booklet because I am old*, *become forgetful*, *and don’t know a lot*.

(Mother, BMC)

Although mothers, as well as fathers, teachers, and male peers, felt it should be the mother’s responsibility to teach and prepare their daughters about menstruation, it was apparent from mothers’ responses that they did not always feel well-informed about M&MHM. Often, mothers had questions about irregular menses, how to care for stomach pain, and how to better dispose of menstrual supplies. Therefore, information about these topics may not have been comprehensively gained by girls in the home environment, even after menarche.

#### Perspectives of fathers

Fathers also acknowledged that lacking important WaSH resources, such as clean water and an acceptable latrine, presented challenges for their daughters, and wives, during menstruation. Fathers also recognized the need for their daughters and wives to have accurate information about MHM:

*[My wife and daughter] lack toilet and water during dry season*. *[They have to] take [a] shower at [a] far place*, *[and there is] no place to change sanitary pad*.

(Father, KT)

*[I want my wife and daughter to have] information about cleaning [their] body*, *[and] what they [need to] do to get healthy*. *[I want them to have] information about women diagnosis because I see some villagers sick*. *So I want them [to] prevent [sickness] and look after their health*.

(Father, BMC)

Recognizing these difficulties, fathers often described wanting to be supportive to their wives and daughters during menstruation, despite some reservations about their role as a male in an otherwise “female matter”. When asked if they felt comfortable assisting their wives or daughters during menstruation, 86.7% (n = 15) of fathers said yes. Fathers described being willing to assist in a variety of ways, with an emphasis on allowing their wives and daughters to rest during menstruation. Fathers also felt these supports helped alleviate FSD for their daughters:

*[I] provide money to buy supply*, *accompany them to hospital*, *help [with] some work at the fields*, *and [I] let my wife and daughter rest at home*.

(Father, BMC)

*[I] help them not to worry*. *[I] help financially*, *accompany to hospital*, *provide soap*, *fetch the water for my daughter and wife*, *[and do] not let them do heavy work*. *But [I] have them take a break for 3–5 days*.

(Father, KT)

Fathers still predominantly felt that menstruation was best attended to by girls’ mothers. Fathers also reported that their daughters did not usually inform them of their MHM needs and that sometimes their daughters’ behavior would change towards them during menstruation, often due to shyness. Fathers also reported trying tried to avoid making their daughters feel uncomfortable by limiting their involvement in the issue:

*The mother is responsible to teach the daughter because she had experience [of menstruation] before*, *and [my] daughter feels comfortable to tell [her] mother about her menstruation*.

(Father, BMC)

*I never tell my daughter because she never talks to me about menstruation*.

(Father, KT)

*Before menstruation*, *I get close to my daughter*, *such as [to] sit and talk*. *After menstruation*, *my daughter feels shy to get closed*, *but we talk as usual*, *[just] not sit close to each other*.

(Father, BMC)

*I didn’t know when my daughter experienced menstruation because I never asked*. *If I ask her*, *she feels shy and thinks that I want to know her personal issue*. *As a father*, *I did not manage this issue*.

(Father, BMC)

These dynamics, therefore, may necessitate the mother taking on a more predominant role. Taken together, paternal involvement that seemed conservative may have been limited in an effort to help alleviate FSD, but not necessarily reflective of not wanting to be more aware and supportive of their daughter’s needs. As one father stated, “Men would like to know how their daughter [is] challenge[d] during menstruation” (BMC).

#### The perspectives of female and male teachers

Both female and male teachers were asked to provide feedback about their awareness of and involvement in girls’ MHM in the school environment, including challenges girls faced at school, whether MHM curriculum existed and how it should be taught, and how challenges to MHM impacted girls’ engagement at school. Female and male teachers often agreed in their feedback concerning these questions during SFGs and SIs. Female and male teachers both recognized the challenges girls faced due to a lack of adequate WaSH resources at their schools. When asked how to improve the situation for girls at school, teachers made several recommendations in keeping with the girls’ responses, adding to them suggestions to have the latrines be separated by gender:

*[At school*, *we should] have enough clean water*, *[*…*] separate toilet[s] for female students*, *soap in the toilet*, *sanitary pad[s] and [a] place for disposal*.

(KT, Female teacher SI)

*[We should] have some supply ready at school (soap*, *sanitary pad*, *tissue*, *tiger balm*, *ointment*, *pain release*, *trash bin inside the toilet*, *bathroom*, *black plastic bag*, *teacher counselors)*.

(KT, Male teacher SI)

SFG Leader: What are some challenges that female students face in managing their menstruation at school with the current toilet facilities?

*Female teacher 3*: *[The] toilet is not clean*, *not enough water*, *[and lacks] soap*.

*Male teacher 2*: *Very few toilets*, *[so we] can’t go when we want*.

*Male teacher 1*: *[We] lack [an] educator on menstrual cycle*.

*Male teacher 2*: *[There is] no medicine to release pain*.

(BMC, SFG n = 5: 3 Female teachers, 2 Male teachers)

The main obstacle teachers discussed to implement these recommendations was lack of budget to purchase new materials and supplies.

Teachers tended to discuss their roles in girls’ menstrual needs as mostly relegated to teaching girls about the importance of hygiene and understanding menstruation as a physical process. When asked about whether any curriculum existed to teach on M&MHM, teachers at each school said there was a curriculum to cover those topics. Both female and male teachers generally agreed that they felt the material was helpful to girls and helped to reduce or alleviate FSD:

*SFG Leader: Have you and female students found the MHM curriculum helpful? Why or why not?*

*All Teachers*: *Curriculum is helpful for student*.

*Female Teacher 7*: *[It helps them] care about hygiene and not [be] smelly at others*.

*Male Teacher 1*: *[We] encourage students during the class to apply after learning*.

*Male Teacher 3*: *After learning*, *the girls won’t be scared to see blood*.

(BMC, SFG n = 7: 4 Female Teachers, 3 Male Teachers)

When teachers were asked whether they felt male and female teachers could teach on the topic of M&MHM, female and male teachers often agreed that both could teach on MHM, though mixed opinions were expressed across schools and within schools. At five of the seven schools included in the study, most teachers felt that both male and female teachers could teach on M&MHM. Between female and male teachers, slightly more female teachers, 82.35% (n = 14/17), reported feeling that both genders could teach on MHM compared to 71.43% (n = 10/14) of male teachers who believed so (Table 8). However, within the same school, teachers did not always reach consensus:

*SFG Leader: Do you think male and female teachers can teach on this topic?*

*Female teacher 1 & 5*, *Male teacher 3*: *Both male and female teachers because they can help the students in some situation*.

*Male teacher 2*: *Both male and female teachers because it is general knowledge*.

*Male teacher 4*: *Female teachers because they are closer to female students*, *and the girls are not shy*.

*Male teacher 6*: *Male and female teachers because some schools don’t have female teachers*, *so male teachers are able to teach also*.

(KT, SFG n = 6: 2 Female Teachers, 4 Male Teachers)

**Table 8:** Teaching beliefs among female and male teachers.

Teaching Beliefs	By Gender	n Responses	N Total Responded	Percentage (n/N)
Believes both female and male teachers can teach about M&MHM	Female	14	17	82.35%
	Male	10	14	71.43%
Believes both girls and boys should be taught about M&MHM together	Female	11	14	78.57%
	Male	11	12	91.67%

Teachers were also asked whether they felt that girls and boys should be taught about MHM together. While 78.57% of female teachers agreed that girls and boys would benefit from being taught together about MHM, slightly more male teachers (91.67%) supported this idea. Of those teachers that did not feel that girls and boys should be taught together, they often cited FSD as a reason that girls and boys should learn separately, stating that girls would feel shy or embarrassed to learn about the topic among male peers. Teachers discussed these views, stating:

*Female teacher 7*: *[We] should separate because the girls feel shy to question in front of the boy*, *[and] some boys mock at the girls*.

*Female teacher 6*: *[We should] not separate because this knowledge should be understood by boys*. *Male teacher 2*: *[We should] not separate because boys have to understand*. *He is able to marry and have children*.

*Male teacher 3*: *[We should] not separate because boy should understand in order to pass on experience to people around [him]*.

(BMC, SFG n = 7: 4 Female teachers, 3 Male teachers)

*Male teacher 1*: *Boys and girls should understand same thing*.

*Female teacher 5*: *[We] should separate because girls can feel shy to ask female teachers*. *[If they] ask more*, *[they] know more*.

*Female teacher 4*: *[We] should not separate because they have equal rights to understand and equality for understanding same thing*.

(BMC, SFG n = 6: 3 Female teachers, 3 Male teachers)

Despite some disagreement about girls’ experience of FSD among male peers if taught together about MHM, both female and male teachers recognized FSD as an underlying reason of why girls would hesitate to reach out to them during times of their menstruation. A few teachers, often female but some male teachers, as well, described giving advice to their female students that was both instructive and comforting, aimed at helping girls resolve FSD surrounding menstruation. However, many teachers, both female and male, reported that besides asking to go home or to use the bathroom, girls often did not ask them for help during menstruation:

*[We] can teach but students dare not to ask in the class*. *[They] keep questions to ask later*.

(BMC, Female teacher SFG)

*Male teachers 2–4 &6*, *Female teacher 5*: *[Girls] never ask for help because feeling shy*.

*Female teacher 1*: *There [*…*] [was] one female student [who] came to ask me [for a] sanitary pad*, *and I gave [it to] her*.

(KT, SFG n = 6: 2 Female teachers, 4 Male teachers)

*[I] never encounter this issue [of helping girls]*. *The students ask their friends to help or help themselves*.

(KT, Female Teacher SI)

Overall, the teachers’ responses reflected many of the girls’ concerns about their MHM experiences at school and their limited support from teachers.

#### The perspectives of boys

Boys were asked about their knowledge of menstruation and their interactions or experiences with girls related to menstruation. Boys expressed that there were several topics that they felt were important for boys to know regarding girls’ menstrual needs, including understanding the basics of menstruation, what girls’ needs are for MHM, and how boys can be supportive. One boy suggested:

*Boys need to know that sanitary pad is important for her*, *and [that] she needs [*…*] [medicine] when she is pain*. *[Boys] need to know about hygiene and health care*.

(Boy SI, BMC, Age 16, Grade 9)

Boys also felt that pragmatic knowledge for girls was important for their menstrual experiences and MHM, as one boy said, “[I] would like women [to] know [how] to clean, use sanitary pad, medicine for pain, not do heavy work and rest more” (BMC, Age 19, Grade 11).

When boys were asked whether they thought they should help girls during menstruation, boys had mixed opinions. Several boys said that they thought they should help girls and gave ideas of how they could assist girls during menstruation:

*[I could] help to buy medicine*, *look after [her]*, *let her get more rest*, *[and] replace and lighten her work like [*…*] schoolwork*.

(Boy SI, BMC, Age 15, Grade 8)

*[I could] help to do housework which is heavy*. *[I could help] wash the dishes and clean the house*.

(Boy SI, KT, Age 15, Grade 8)

*[I] should help because they are my friend*. *And I get to know when my younger sister [is] having menstruation*, *she is uncomfortable*, *same as other people*.

(Boy SI, BMC, Age 16, Grade 8)

*[Boys] should help*. *[I] encourage*, *not mock [or] tease her*. *[I try to] be happy with her when she [is] experiencing menstruation to avoid [being] offended by me*.

(Boy SI, BMC, Age 20, Grade 12)

However, some boys said they did not think they should help girls, either because they did not know how or because they were concerned about upsetting the girls. Boys also expressed feeling shy of approaching or interacting with girls during menstruation, either because they did not want to upset girls or did not understand the girls’ needs at that time:

*I want to help but I dare not because the girls and I are shy*.

(Boy SI, KT, Age 15, Grade 9)

*I don’t know how to help or what to do*.

(Boy SI, KT, Age 17, Grade 9)

*[Boys] should not help because it can influence her [to be] shy*. *Only women can understand and help each other*, *but men can talk a bit about how to care the health*.

(Boy SI, BMC, Age 17, Grade 11)

*[When a] girl becomes mature*, *I dare not to get close because we [are] shy [with] each other*. *We are close before because we play[ed] like brother and sister*.

(Boy SI, BMC, Age 17, Grade 11)

When boys were asked what improvements could be made at school or home to help girls have better menstrual experiences and MHM, some boys gave thoughtful responses, saying:

*[It is important to] encourage the girls not to [be] scared[d] [or] shy*. *School should have toilet for girls or women*, *clean water*, *warm water*, *educate about menstrual hygiene*, *and have pain killer sometimes*. *Family should pay attention [to] girls [and] can prepare medicine*, *warm water and sanitary pad*.

(Boy SI, BMC, Age 17, Grade 12)

*[The school] should have female and male toilet separated*, *clean water*, *and help to educate them*. *School should have sanitary pad for women use*.

(Boy SI, BMC, Age 17, Grade 11)

*The school should explain about menstrual hygiene*. *Home should have toilet (have toilet supply)*, *water inside the toilet*, *and [a] jar for cleaning during menstruation and cleaning house*.

(Boy SI, BMC, Age 19, Grade 9)

*School should have medicine for female students when they have problem with headache or stomach pain during menstruation*. *[School] should have sanitary pad and clothes to change when they [are] stained*.

(Boy SI, BMC, Age 20, Grade 12)

Some boys admitted that they did not know what would help girls at school or at home during menstruation. Nevertheless, although boys were not routinely thought of as comforting to girls during menstruation, it appears that some boys have been thoughtful enough to recognize ways to improve girls’ experiences. Taken together, it was apparent from girls’ and boys’ responses that there is presently a lack of communication about M&MHM often due to FSD among both girls and boys.

### Impact of FSD on girls’ behavior

When asked about how their M&MHM experiences impacted their participation in school or in communal activities, several girls indicated that times of menstruation contributed to disruption in their regular activities and engagement. Fifty percent (n = 37/74) of girls said FSD and/or corresponding M&MHM challenges caused them to miss communal activities, such as attending weddings, visits to neighbors or friends, activities working in the field, and general household activities during menstruation, often due to FSD about staining, odors, or lack of resources.

*[I] do not go [to] other’s house because I am afraid that someone see [referring to knowing it’s her menstruation]*, *and sometimes the girls forget there is blood staining on clothes*. *[It] feels hard*, *and [there is] back pain for each menstruation*.

(Girl SI, BMC, Age 16, Grade 8)

*[I] missed going to [a] wedding reception because [I was] afraid of being smelled and stained*.

(Girl SI, KT, Age 16, Grade 9)

*[I] should not clear the grass at the cassava plantation*, *transplant at the rice field*, *[or] go out to the neighbor’s house because it is shame[ful] if [I am] [*…*] seen [with] the stain when [doing] transplantation or clear the grass*.

(Girl SI, KT, Age 16, Grade 8)

*[I] missed my friend[‘s] launching house*, *some celebration*, *[and I do] not do housework because feeling stomach pain [and they a] lack clean toilet for washing at place of celebration*.

(Girl SI, BMC, Age 18, Grade 8)

In such cases, FSD and/or a lack of resources were contributing to whether girls felt safe and comfortable attending otherwise favorable or routine activities. Due to issues associated with sanitary pads not providing adequate protection against leakage and stains, girls also described transit between locations (e.g., home to school, home to the market) being difficult and related to FSD:

*[I] should not walk far*, *sit [for a] long [time]*, *[or] ride motor to market because [this] can get us wet or cloth stain that people can see*.

(Girl SI, KT, Age 15, Grade 8)

*[During menstruation*, *it is] difficult to sit when bleeding too much*, *[*…*] to come to class by the bike or motor*, *[or] [*…*] to do heavy work because stomach pain*.

(Girl SI, BMC, Age 15, Grade 9)

These concerns in transit before girls arrived at school meant they were already experiencing FSD before having to navigate the additional MHM challenges of the school environment.

Thirty-six percent (n = 27/75) of girls reported missing school-days due to M&MHM challenges, and 37.3% (n = 28/75) of girls said their participation in class was affected. One girl described her experience, stating, “[At school, I feel] bored, afraid of being seen and shy, no mood to study. I want to go home and want to be alone” (BMC, Age 17, Grade 11).

Despite this, eighty-four percent (n = 63/75) of girls said their menstruation did not impact their ability to take exams, and the general feedback was that exams were important to academic success, even when managing their M&MHM was difficult, as one girl described:

*Because I think that menstruation of women is normal*. *Even [if] I feel stomach pain a little during menstruation*, *[*…*] I have to come because exam is important*.

(Girl SI, KT, Age 16, Grade 9)

If girls did choose to miss an exam due to menstrual challenges, girls would ask for supplementary exams, as needed. Teachers were generally understanding if a girl needed to return home due to menstrual challenges, but they sometimes shared that they did not think girls did as well when making up their exams.

## Discussion

While Cambodia’s government has considered developing a new standard for the number of students per latrine in schools [15], the National Ministry of Education, Youth, and Sport and the National Ministry of Rural Development did not have action plans to address MHM at the school or household level at the time of this study [21]. Our findings confirm that rural Cambodian girls’ M&MHM experiences are impacted by managerial, knowledge-related, and social factors, which could either be supportive to girls’ MHM or pose challenges during menstruation. When girls encountered MHM challenges due to lack of managerial resources, adequate MHM knowledge, or reassuring social support, girls described experiencing fear, shyness, and discomfort (FSD) during their menstruation. Prior studies in countries such as Zambia, Kenya, Ethiopia, Tanzania, Ghana, India, and Lao have also identified FSD as a widespread concern for many adolescent girls associated with M&MHM challenges [4, 8, 9, 13, 16, 17]. The girls’ individual reported MHM outcomes and experiences of FSD were influenced by supports and challenges on multiple levels of the SEM framework, such that interpersonal, communal, and societal level vulnerabilities and strengths directly impacted girls’ perceived self-efficacy and ability to navigate their MHM.

At an individual level, girls described having to be resourceful to manage their MHM and avoid FSD related to menstrual accident by securing menstrual supplies and information as best they could, often dependent upon their available interpersonal, communal, and societal level resources. Interpersonally, girls sought to navigate parental, teacher, and peer relationships in ways that alleviated FSD and enhanced MHM during menstruation, at times by reaching out for help and at times by avoiding interaction altogether. Communally, both villages and schools faced varying levels of access to WaSH resources, such as clean water and acceptable latrines, which girls and women need during their menstruation for a variety of hygiene practices. Challenges at this level had varying impacts on girls’ behavior and decision-making and influenced their school engagement and participation in household or communal activities. Communal level challenges to accessing these valuable WaSH amenities often reflected the societal level challenges to provide adequate coverage of these resources to households and schools within Cambodian rural provinces. As such, interventions aiming to address girls’ MHM needs as a means of supporting girls’ dignity, education, and navigation into adulthood would need to assess pragmatic MHM supports along these multiple levels and understand how FSD influences girls’ receptivity to MHM-related interventions while also normalizing girls’ MHM needs.

In this study, girls’ experience of FSD in social engagements at home or in school was often influenced by their ability to address managerial challenges and comfortably practice MHM, whether in their homes, villages, or at school. Girls experienced vulnerabilities in being able to successfully practice MHM due to managerial stressors such as unreliable sanitary pads, unsatisfactorily maintained latrines, lack of clean water and soap, and the absence of private disposal options, all of which increased FSD. When girls could not find acceptable ways to navigate these stressors, their MHM practices and perceived self-efficacy related to MHM were undermined. Many girls expressed greater comfort and confidence in navigating MHM at home. In the home environment, parents, particularly mothers, sought to help girls overcome managerial challenges and alleviate FSD by providing supplies, information, and encouragement. Girls rarely discussed their fathers’ support during menstruation, but paternal support was often implied in the provision of supplies by their “parents”. Mothers and fathers affirmed that role, but fathers offered additional insight into the ways they attempted to help their wives and daughters during menstruation. Fathers were clearly concerned about their wives’ and daughters’ overall health and often expressed not wanting to add to their daughter’s FSD during menstruation. Fathers felt this could best be achieved by respecting their daughters’ privacy and enabling them to rest more when they were aware of their daughters’ menstruation. Fathers often expressed having limited knowledge in the area of menstruation, thus transferring more responsibility to their wives. However, other studies have found that when fathers are educated and involved, there can be enhanced support for women in the household through increases in household budgets for sanitary materials and enhanced paternal motivation to create WASH infrastructure considerate of MHM needs [25].

Girls’ FSD at school due to fears of staining, insecurity about odors, and shyness around boys was increased by managerial challenges (e.g., unmaintained latrines, lack of MHM emergency supplies, etc.). Female peers often played a key role in supporting each other at school by helping each other find menstrual supplies or covering up each other’s menstrual accidents if they occurred due to lack of preparation or supplies. These interactions between managerial challenges, social experiences, and MHM practices suggest that addressing girls’ practical MHM needs to reduce their managerial challenges could foster greater confidence and reduce social FSD, particularly at school. Transitioning these vulnerabilities concerning managerial challenges into strengths that improved girls’ MHM and reduced FSD at home and at school would necessitate interventions that increased access to WaSH resources such as clean water and latrines, provided girls with routine and emergency access to menstrual supplies (i.e., pads), and considered girls’ concerns for privacy when needing to clean themselves or dispose of menstrual supplies. Rural Cambodian schools and households often face financial and logistical barriers to improving their access to clean water, improved WASH facilities, and quality MHM supplies. Governmental and NGO support to improve access to these resources in schools and villages could reduce these challenges.

Many Cambodian girls in this and prior studies [2, 15, 16] indicated gaps in M&MHM knowledge that increased FSD. Several girls expressed having had little to no biological or practical knowledge about menstruation prior to menarche, leading girls to fear that they were experiencing a negative health outcome or sickness. FSD due to lack of knowledge led some girls to hide their menarche, which precluded more immediate support and explanation of their experience. Girls needed interpersonal support to be provided with timely and reliable M&MHM knowledge, with particular attention to pragmatic information, such as how to properly wear sanitary pads and practice good hygiene. As in our study, Sommer et al. also found that Cambodian girls often sought assistance and information from their mothers, as well as from sisters and other female relatives, but mothers were not always adequately preparing their daughters for menstruation prior to the onset of menarche [16]. Similar circumstances have been found in other contexts, such as among rural Indian adolescent girls, who found their mothers to be supportive, but also ineffective, when it came to decision-making about menstrual health-related challenges [26].

Although some girls clearly recalled having information provided to them at school, other girls at the same school would sometimes report receiving no information, suggesting that the information shared may not have been effectively delivered to engage all girls in need of menstrual guidance. Girls reported preferring to learn about M&MHM from a booklet for reasons of privacy; their mothers, due to relational closeness and prior experience; or female teachers, who were thought to be educated and could empathize as a woman, but male teachers were also considered as resources. While teachers recognized the importance of MHM education for girls, they also reported having questions about M&MHM, suggesting that the MHM curriculum cited may not have been a sufficient source of M&MHM information.

Considering the vulnerabilities girls faced to FSD and delayed menstrual understanding due to lack of M&MHM knowledge, it would be important to support girls through enhanced M&MHM education and increased interpersonal support. MHM knowledge about how to properly wear sanitary pads was of particular interest to rural Cambodian girls. Girls who did not understand proper sanitary pad use often experienced increased FSD due to fears of leakage and staining. Similar challenges have been reported in other contexts, such as among Ghanaian schoolgirls [27]. However, a “pads-with-education” intervention among Ghanaian schoolgirls found that when girls were provided with sanitary pads, education on puberty, and education on how to properly use sanitary pads, they experienced increased MHM knowledge, self-efficacy, and a 9% increase in school attendance compared to girls who did not receive all three components [27]. Sommer et al. also demonstrated that using booklets to teach girls about M&MHM was highly effective in Tanzania [16], and this could be effective in the rural Cambodian context given the number of girls expressing a preference for learning from a booklet for privacy. Some parents, teachers, and male peers also expressed interest in better understanding girls’ M&MHM needs to provide interpersonal support. Including girls’ parents, educators, and male peers in educational interventions that identified and supported girls’ M&MHM needs could benefit girls further by increasing the awareness and understanding of their social supports.

Girls’ FSD related to experiencing their menstruation around male peers was also connected to a fear of being teased or “talked about” if their male peers knew they were experiencing menstruation, especially due to a menstrual accident. In other contexts, a few studies have demonstrated that MHM education is very important for both adolescent girls and boys in the school environment to normalize menstruation and reduce FSD and stigma. Play-based education, where students are introduced to new ideas and concepts through games and interactive activities, has proven effective in MHM education among girls and boys. One study that examined the effect of using a play-based approach to introduce MHM into Ghanaian school curricula showed that teacher engagement in the activities about menstrual knowledge resulted in girls being less shy to discuss menstrual needs with them at school [28]. Dorgbetor et al. found that both girls and boys willingly participated with interest in the MHM related activities, showing the effectiveness of the play-based approach to overcome FSD [28]. Following these programming activities, girls and boys displayed greater confidence in openly discussing menstrual related experiences together without FSD [28]. Teachers who participated in the play-based study also found that after educating girls and boys together about menstruation, boys refrained from teasing girls and instead would respectfully and politely engage with girls about MHM [28]. Similar behavioral modifications among boys, after educating them about menstruation, have also been shown in Kenya, where boys subsequently made greater efforts to help clean school latrines following education [29]. These benefits even extended into the home environment, as boys also reported helping the women in their families with duties they would not have normally done at home [29]. Given these benefits, it will be important to determine acceptable, non-embarrassing methods to educate boys and girls about menstruation, potentially at the same time.

This and prior studies [17] have shown that MHM challenges and FSD associated with menstruation can impact girls’ participation in school or communal activities. The structured interviews and focus groups used for this study’s data collection yielded valuable insights about those challenges, as well as girls’ resilience and resourcefulness to better navigate their M&MHM. When considering the limitations of this study, a semi-structured interview approach could have enabled interviewers to explore topics more deeply and gain additional insights. By using enumerators, rather than voice-recording and transcription, the loss of some participant ‘voice’ may have curtailed nuances not captured during enumeration. However, the consistency of described experiences across participant groups and different enumerators is supportive of the data being reflective of participants’ experiences. Additionally, discomfort for some participant groups, such as boys and fathers, in discussing menstruation may have limited participant responses. To mitigate this, an all-female team performed data collection among female participants, and cross-gender conversations were treated with respect and openness for questions throughout the interviews. A further limitation relates to respondent selection. Most of the respondents (57%) were randomly invited from class lists. Only six of the randomly invited students returned opt-out forms, which suggests a good random sampling within the student population. The mothers (28% of respondents) and father (4% of respondents) were selected by the village chief or community leader. Teachers, who volunteered to participate (11% or respondents) largely self-selected, which likely introduced some selection bias. Finally, a limitation of the study relates to the rural setting of the study, in selected schools in two largely rural provinces in Cambodia. Given that the sample population excluded urban dwellers, the results should not be generalized to experiences of adolescent girls in urban settings in Cambodia.

Based on girls’ experiences and participant recommendations, interventions that aim to reduce FSD and enhance social, educational, and managerial resources comprehensively, rather than addressing managerial resources only, could prove effective in reducing FSD, resolving barriers to effective MHM practices, and advancing girls’ ability to reach their full social and educational potential across environments. Comprehensive strategies could incorporate a “FSD rapid test” to gauge girls’ experiences of FSD before and after new M&MHM interventions as a measure of intervention effectiveness; employ creative educational campaigns that include parents, teachers, and male and female youth; and aim to provide key WaSH resources, such as improved latrine designs, access to clean water, and greater access to quality menstrual materials (e.g., pads). To further promote increased comfort for girls at school, creative learning approaches such as ‘play-based’ education can be implemented to improve the interactions between both girls and teachers and between girls and boys regarding M&MHM. Tailoring comprehensive strategies to the national and local contexts in Cambodia or other settings would provide holistic approaches to improving M&MHM experiences and outcomes for girls.

## Conclusions

The findings of this study have served to validate prior evidence on the challenges faced by rural Cambodian adolescent girls in relation to M&MHM, with an expanded scope inclusive of both the home and school environments. Based on girls’ descriptions of their menstrual experiences across environments, it was clear that FSD had a significant impact on girls’ behavior, confidence, and decision-making. FSD was associated with relational discomfort, lack of access to key MHM facilities and resources, and gaps in knowledge that impacted the pragmatic aspects of MHM. Often underlying MHM pragmatism was the need for reliable, high-quality sanitary pads that would adequately meet girls’ needs across environments and in transit. Without better quality resources, girls will continue to face ongoing challenges with FSD. Affirming and resourceful relationships, comprehensive and timely knowledge of M&MHM, and access to MHM-optimized WASH facilities helped to alleviate FSD, as girls emphasized needing these aspects to overcome FSD and associated barriers.

The authors hypothesize that given the appropriate supports, girls will experience less FSD, which will reduce the critical tensions girls face relationally, educationally, and contextually during menstruation. Since FSD also contributed to lower hygienic standards for MHM, alleviating FSD may promote better menstrual decision-making, thus leading to improved menstrual, relational, educational, and health-related outcomes among adolescent girls. Supporting the dignity of adolescent girls must include efforts to eliminate FSD associated with menstruation. Reductions in FSD should be a key indicator as to the success of future interventions. Future research should continue to explore MHM in multiple environments (e.g., home, workplace) to determine underlying factors that contribute to FSD. Further evidence regarding the specific challenges that caregivers face in supporting girls during menstruation across environments will also provide valuable knowledge on how to design interventions towards having a generational impact on reducing FSD.

Although MHM is often framed as a women’s and girls’ issue, it is important to consider the impact FSD has on family cohesion between parents and daughters, school experiences between teachers and female students, as well as in the social dynamic between boys and girls. As such, addressing the commonly unserved needs of women and girls regarding MHM and FSD will serve to promote the confidence and comfort of everyone involved. Stakeholders from government agencies, NGOs, and industry can prioritize addressing factors that underlie FSD and its impact on girls’ MHM outcomes. Through interdisciplinary efforts, the Cambodian Ministry of Education, Youth and Sport, the Ministry of Health, and the Ministry of Rural Development can incorporate the recommendations identified through this study into their future WaSH initiatives.

## Supporting information

S1 File Inclusivity in global research. (DOCX)

S1 Data (XLSX)
