# Supplementary material for: Navigating fear, shyness, and discomfort during menstruation in Cambodia
Source: PLOS Glob Public Health. 2022 Jun 9;2(6):e0000405. doi: 10.1371/journal.pgph.0000405 (PMC10022126; doi:10.1371/journal.pgph.0000405)
Supplement: S1 File — (DOCX) [file pgph.0000405.s001.docx]

Inclusivity in global research

PLOS’ policy on inclusivity in global research aims to improve transparency in the reporting of research performed outside of researchers’ own country or community and ensures that PLOS publications reporting global research adhere to high standards for research ethics and authorship. Authors of relevant research articles may be asked to complete the questionnaire below, which outlines ethical, cultural, and scientific considerations specific to inclusivity in global research. This questionnaire may be requested when researchers have travelled to a different country to conduct research, if research uses samples collected in another country, research with Indigenous populations or their lands, or if research is on cultural artefacts. Researchers travelling to another country solely to use laboratory equipment will not normally be required to complete the questionnaire. However, the questionnaire can be requested at the journal’s discretion for any submission – if you have been requested to complete this questionnaire by the PLOS journal you submitted to, please do so.

Please complete the questionnaire below and include this as a Supporting Information file with your manuscript. Note that if your paper is accepted for publication, this checklist will be published with your article in the supporting information files. Please ensure that you reference the checklist in the main body of your manuscript. We suggest adding a subsection ‘Inclusivity in global research’ to your Methods section and adding the following sentence: “Additional information regarding the ethical, cultural, and scientific considerations specific to inclusivity in global research is included in the Supporting Information (SX Checklist)”

The questions have been designed to be applicable to a wide range of study types, and there are subsections for both human subjects research and non-human subjects research. If any of the questions are not relevant to your research please mark them as “N/A” as appropriate.

**Ethical considerations, permits and authorship**

*This section is applicable to all research types.*

Provide details as to who granted permissions and/or consent for the study to take place in the Methods section of your manuscript. This should include the names of **all** ethics boards, governmental organizations, community leaders or other bodies that provided approval for the study. If individuals provided approval refer to these people by their role or title but do not list their name(s).

Reported on page number: On page 9: “Ethical approval for this study was granted from the institutional review board of Yale University’s Human Subjects Committee (HSC Protocol 1506016029; Approval June 12, 2015) and from the Cambodian National Institute of Public Health’s National Ethics Committee for Health Research (236 NECHR, Approval July 3, 2015).”

If there were any deviations from the study protocol after approval was obtained please provide details of these changes in the Methods section of your manuscript.

Reported on page number: On page 6: “Parents were eligible if they were 40 years or older and had at least one daughter who was 14 years old, although the protocol was amended to include younger parents aged 30 years or older so that younger mothers who had traveled long distances, or who were particularly interested in learning from the focus group discussions, could also participate.”

Did this study involve local collaborators that are residents of the country where the research was conducted or members of the community studied? If you do not have any authors from said communities, please provide an explanation for this below. Everyone listed as an author should meet PLOS’ criteria for authorship and all individuals who meet these criteria should be included in the author byline, rather than the acknowledgements. Authorship criteria is based on the International Committee of Medical Journal Editors (ICMJE) Uniform Requirements for Manuscripts Submitted to Biomedical Journals - for further information please see here: <https://journals.plos.org/plosone/s/authorship>.

Per page 8 of the revised manuscript: “The research team consisted of the primary investigator, two Cambodian women as team leaders, and seven young Cambodian women serving as enumerators, all of whom were trained in study methods to understand the questions and engage participants.” The two Cambodian team leaders are recognized in the acknowledgement section for their contribution to the data collection and field component. Their scope of contribution did not include three of the four ICMJE conditions for authorship credit. They did not contribute to drafting the article, review of the manuscript, or agreement to be accountable for the accuracy and integrity of the work.

The five listed co-authors meet the four ICMJE conditions for authorship credit.

**Human subjects research (e.g. health research, medical research, cross-cultural psychology)**

Did you obtain written informed consent from a representative of the local community or region before the research took place? How did you establish who speaks for the community? Details of written informed consent obtained from study participants should be reported separately in the Methods section of your manuscript.

From page 8 of the revised manuscript: “Approval to engage with the communities was sough in the advance from the Provincial Department of Rural Development for both BMC and KT. In addition, approval for activities at the schools was sought from the Provincial Department of Education, Youth and Sport for both BMC and KT.”

How did members of the local community provide input on the aims of the research investigation, its methodology, and its anticipated outcome(s)?

The aims of the research investigation were developed over a number of years of community engagement on the part of Samaritan’s Purse. In this way, the community had some indirect input. In addition, the Cambodian Water, Sanitation and Hygiene working group was consulted on the research intentions and methodologies ahead of data collection.

When engaging with the local community, how did you ensure that the informed consent documents and other materials could be understood by local stakeholders?

From pages 8 and 9 of the revised manuscript: “All participants provided informed consent prior to enrollment, and all interactions between team leaders and potential participants were conducted in Khmer.

Team leaders reviewed the consent forms verbally in Khmer that explained the study’s topic of MHM with all potential participants prior to receiving their consent. Adults gave verbal consent prior to being interviewed or participating in a SFG. For minors under 18 years of age, they were also provided with a parental opt-out form in Khmer, which was either given directly to the students by the research team or distributed to the students by the school director. Students were asked to discuss the parental opt-out form with their parents. The parental opt-out forms were to either be signed and returned, signifying that the student could not participate, or kept at home as indication of consent.

Team leaders verbally reviewed the study eligibility and purpose with girls and boys who had permission to participate, or who were old enough (18 years old or older) to consent themselves, before beginning the SFGs and SIs. The girls and boys had the opportunity to decline participation at that time or at any point during the conversation due to the sensitive nature of the topic. Only six students returned their parental-opt out forms, indicating a lack of parental consent to have their child participate, and those students were not included in the study.”

Will the findings of the research be made available in an understandable format to stakeholders in the community where the study was conducted (e.g. via a presentation, summary report, copies of publications, etc.)? Please provide details of how this will be achieved.

The preliminary findings were presented to the National Water and Sanitation Working Group and to the Provincial Departments of Rural Development for both BMC and KT provinces. In addition, copies of the publication will be provided to the National Water and Sanitation Working Group as well as Cambodian National Institute of Public Health’s National Ethics Committee for Health Research as a condition of the approval the committee provided.

**Non-human subjects research using specimens/ animals collected as part of the study, or those housed in archival collections. Examples include archaeology, paleontology, botany and zoology.**

Did the permission you obtained from a local authority to perform the study include an agreement on access to outputs and benefit sharing? This may include procedures to enable fair distribution of the benefits and resources arising from the research performed. Please include any details of Prior Informed Consent and Benefit Sharing Agreements obtained. These may be required by field-specific regulations, for example the Convention on Biological Diversity (CBD) and the associated Nagoya Protocol.

There were no non-human subjects for this study.

If the material used in your study was imported, please A) provide the year it was imported and B) indicate whether permits were obtained to import/export the materials used, C) provide details of any permits obtained. If this information is not available, please indicate this.

There were no materials imported for this study.

If you used archival specimens, please state how the material used in your study was acquired by the institute it is held in and provide details of any permits obtained for the original excavations/ sample collection. If this information is not available, please indicate this.

There were no archival specimens used in association with this study.

How was the potential cultural significance of the materials collected in your study to local communities considered in your research design? Were Indigenous peoples and/or local researchers and institutions involved with archaeological excavations / collection of specimens? If so, please provide a description of their involvement.

No materials of potential cultural significance were collected for this study.

If your manuscript includes photographs of human remains please indicate whether authors obtained permission from descendants or affiliated cultural communities to do so.

The manuscript does not include any photographs.
